# Supplementary material for: Identification of photoperiod-induced specific miRNAs in the adrenal glands of Sunite sheep (Ovis aries)
Source: Front Vet Sci. 2022 Jul 22;9:888207. doi: 10.3389/fvets.2022.888207 (PMC9354845; doi:10.3389/fvets.2022.888207)
Supplement: Supplementary file 1 [file Data_Sheet_1.ZIP › Supplementary file/miRDeep2 file.zip!/miRDeep2 file/Know_Structure/SP42A1/oar-let-7a.pdf]

miRBase precursor : oar-let-7a
Total read count : 215708
oar-let-7a read count : 214229
remaining reads : 1479

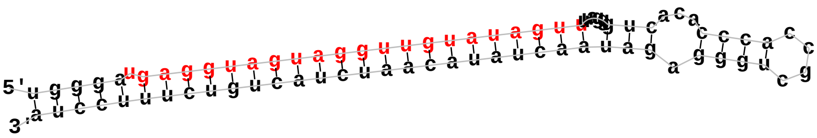

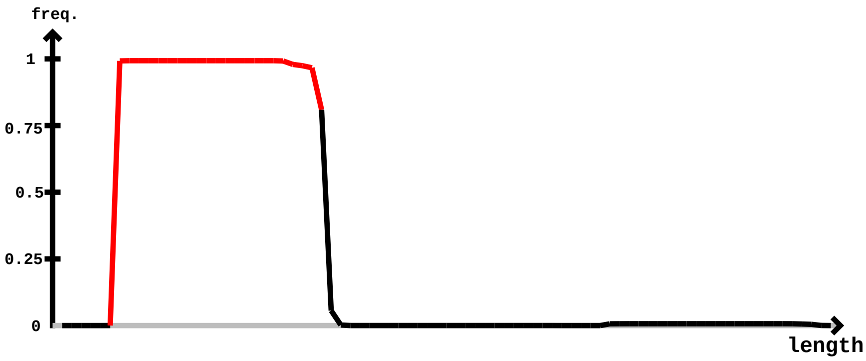

oar-let-7a

5'- ugggaugagguaguagguuguauaguuuuagggucacacccaccgcugggagauaacuauacaaucuacugucuuuccua -3' exp

(((((.(((((((((((((((((((((.....(((...((((....)))).)))))))))))))))))))))))))))))	reads	mm	sample
....augagguaguagguuguauagu..	2	0	seq
....Uugagguaguagguuguauagu..	2	1	seq
....augagguaguagguuguauaguu...	9	0	seq
....Uugagguaguagguuguauaguu...	4	1	seq
....augagguaguagguuguauaguuu..	1	0	seq
.....ugagguUguagguugua..	1	1	seq
.....ugagguagGagguugua..	22	1	seq
.....uUagguaguagguugua..	1	1	seq
.....ugagguaguaAguugua..	2	1	seq
.....ugagguaguagguugua..	210	0	seq
.....ugaAguaguagguuguau...	13	1	seq
.....ugagguagGagguuguau...	255	1	seq
.....ugagguaguagguuguau...	2455	0	seq
.....ugagguaguagguuUuau...	16	1	seq
.....ugagguUguagguuguau...	7	1	seq
.....ugagguaguaAguuguau...	14	1	seq
.....ugagguagGagguuguaua..	124	1	seq
.....ugagguaguagguuguaua..	916	0	seq
.....ugagguagGagguuguauag...	256	1	seq
.....ugagguaguagguuguauag...	1436	0	seq
.....ugagguagGagguuguauagu..	4379	1	seq
.....ugagguaguagguuguauagu..	29550	0	seq
.....ugagguaguagguuguauaguu...	148330	0	seq
.....ugagguagGagguuguauaguu...	13996	1	seq
.....ugagguaguagguuguauaguuu..	11637	0	seq
.....ugagguaguagguuguauaguuuA...	64	1	seq
.....ugagguaguagguuguauaguuuu...	373	0	seq
.....ugagguaguagguuguauaguuuua..	3	0	seq
.....ugagguaguagguuguauaguuuuagggu..	1	0	seq
......gagguaguagguuguau...	1	0	seq
......gagguaguagguuguauag...	1	0	seq
......gagguaguagguuguauagu..	23	0	seq
......gagguagGagguuguauagu..	4	1	seq
......gagguaguagguuguauaguu...	97	0	seq
......gagguagGagguuguauaguu...	10	1	seq

oar-let-7a

```
ugggaugagguaguagguuguauaguuuuagggucacacccaccgcugggagauaacuauacaaucuacugucuuuccua
......gagguaguagguuguauaguuu....................................................    6     0     seq
.......agguaguagguuguauagu......................................................    1     0     seq
.......agguaguagguuguauaguu.....................................................    3     0     seq
........gguaguagguuguauaguu.....................................................    1     0     seq
.........guaguagguuguauaguu.....................................................    3     0     seq
..........uaguagguuguauaguu.....................................................    1     0     seq
...........................uuagggucacacccaccgcuggg..............................    2     0     seq
...........................uuagggucacacccaccgcuggga.............................    13    0     seq
...........................uuagggucacacccaccgcugggaga...........................    1     0     seq
...........................uuagggucacacccaccgcugggagau..........................    1     0     seq
...................................................gauaacuauacaaucuacugucuu.....    1     0     seq
.....................................................uaacuauacaaucuacugucuu.....    2     0     seq
.....................................................uaacuauacaaucuacugucuuu....    4     0     seq
........................................................cuauacaaucuacugucuu.....    4     0     seq
........................................................cuauacaaucuacuguUuuu....    1     1     seq
........................................................cuauacaaucuacugucuuu....    204   0     seq
........................................................cuauacaaucuacugucuuuc...    248   0     seq
........................................................cuauacaaucuacuguUuuuc...    1     1     seq
........................................................cuauacaaucuacugucuuucc..    903   0     seq
........................................................cuauacaaucuacugucuuuccu.    37    0     seq
.........................................................uauacaaucuacugucuuu....    1     0     seq
.........................................................uauacaaucuacugucuuuc...    2     0     seq
.........................................................uauacaaucuacugucuuucc..    14    0     seq
.........................................................uauacaaucuacugucuuuccu.    29    0     seq
..........................................................auacaaucuacugucuuuccu.    10    0     seq
```
